# Effectiveness of the hypotension prediction index in non-cardiac surgeries: a systematic review, meta-analysis and trial sequential analysis

**DOI:** 10.1016/j.bjane.2025.844649

**Published:** 2025-06-10

**Authors:** Vitor Alves Felippe, Ana C. Pinho, Lucas M. Barbosa, Ivo Queiroz, Arthur H. Tavares, Rodrigo Diaz, Carlos Darcy Bersot, Jean-Louis Vincent

**Affiliations:** a Instituto Nacional de Câncer (INCA), Rio de Janeiro, RJ, Brazil; b Universidade Federal de Minas Gerais, Departamento de Medicina, Belo Horizonte, MG, Brazil; c Universidade Católica de Pernambuco, Departamento de Medicina, Recife, PE, Brazil; d Universidade de Pernambuco, Departamento de Medicina, Recife, PE, Brazil; e Universidade Federal do Rio de Janeiro, Hospital Universitário Clementino Fraga Filho, Rio de Janeiro, RJ, Brazil; f Escola Paulista de Medicina da Universidade Federal de São Paulo (EPM-UNIFESP), Programa de Pós-Graduação em Medicina Translacional, São Paulo, SP, Brazil; g Université Libre de Bruxelles, Erasme University Hospital, Brussels, Belgium

**Keywords:** Hypotension prediction index, Intraoperative hypotension, Meta-analysis, Non-cardiac surgery, Randomized controlled trials, Trial sequential analysis

## Abstract

**Background:**

The efficacy of the Hypotension Prediction Index (HPI) for reducing Intraoperative Hypotension (IOH) among patients undergoing non-cardiac surgeries remains unclear. We aimed to perform a systematic review, meta-analysis, and trial sequential analysis to determine whether the HPI is effective for adult patients undergoing non-cardiac surgeries. This study was prospectively registered in the PROSPERO database (CRD42024571931).

**Methods:**

PubMed, Embase, and Cochrane were systematically searched for Randomized Controlled Trials (RCTs) comparing HPI-guided therapy with standard care in non-cardiac surgeries. We computed Mean Difference (MD) and Risk Ratios (RR) for continuous and binary outcomes, respectively, with 95 % Confidence Intervals (95 % CI). Statistical analyses were performed using R Software, version 4.2.3.

**Results:**

We included 11 RCTs, comprising a total of 789 patients, of whom 395 (50.1 %) received HPI-guided management. HPI significantly reduced the Time-Weighted Average (TWA) of Mean Arterial Pressure (MAP) < 65 mmHg (MD = -0.23 mmHg.min^-1^; 95 % CI -0.35 to -0.10; *p* < 0.01) and the Area Under the Curve (AUC) of MAP < 65 mmHg (MD = -97.2 mmHg.min^-1^; 95 % CI -143.4 to -50.98; *p* < 0.01). HPI also decreased the duration of MAP < 65 mmHg (MD = -16.22 min; 95 % CI -25.87 to -6.57; *p* < 0.01) and the number of hypotensive episodes per patient (MD = -3.38; 95 % CI -5.38 to -1.37; *p* < 0.01). No significant differences were observed in the number of hypotensive events, phenylephrine use, or AKI incidence (*p* > 0.05).

**Conclusion:**

In adult patients undergoing non-cardiac surgeries, HPI use was associated with a reduction in the duration and severity of IOH, with no significant difference for adverse events. Limitations include significant heterogeneity across studies, differences in HPI implementation, and lack of long-term outcome data.

## Introduction

Intraoperative Hypotension (IOH) is a common and serious complication during surgical procedures, characterized by a significant drop in blood pressure.^1^ Inadequate management of IOH can lead to detrimental effects such as organ dysfunction, prolonged hospital stay, and increased mortality.^2^^,^^3^ Therefore, ensuring hemodynamic stability is essential, particularly considering recent rapid recovery protocols that aim to minimize the impact of hypotension on patient outcomes.^4^

Current strategies for managing IOH primarily rely on standard hemodynamic monitoring techniques, such as intermittent blood pressure measurements and continuous monitoring with or without advanced cardiac output measurements.^5^ However, these methods are inherently reactive, responding to hypotensive episodes only after they occur. This reactive nature often results in delayed interventions and potentially preventable complications.^6^ To overcome these limitations, the Hypotension Prediction Index (HPI), commercially developed by Edwards Laboratories, provides a preemptive approach to hypotension management by predicting and preventing hypotensive events before they are consistent.^7^^,^^8^

HPI systems work by analyzing over 2.6 million features from a single arterial waveform and comparing them to 133 million waveform patterns to predict hypotensive events. This comprehensive monitoring capability allows the HPI system to provide continuous predictive insights and early warnings of potential IOH up to 15 min before the event with high sensitivity and specificity.^9^ Several Randomized Controlled Trials (RCTs) have demonstrated that HPI-guided monitoring can effectively reduce the duration and severity of hypotensive episodes compared to standard monitoring practices.^10^, ^11^, ^12^, ^13^ Therefore, we aimed to perform a systematic review, meta-analysis, and trial sequential analysis to compare the efficacy of HPI versus standard monitoring in patients undergoing non-cardiac surgeries compared to standard hemodynamic monitoring.

## Methods

This systematic review and meta-analysis was conducted following Cochrane recommendations and Preferred Reporting Items for Systematic Review and Meta-Analyses (PRISMA) guidelines.^14^^,^^15^ The study protocol was prospectively registered in the International Prospective Register of Systematic Reviews (PROSPERO) database under protocol number CRD42024571931.

### Eligibility criteria

Inclusion in this meta-analysis was restricted to studies that met the following eligibility criteria: (I) RCT; (II) Among adult patients undergoing non-cardiac surgeries; (III) Comparing HPI with standard monitoring; and (IV) Reporting at least one outcome of interest. Exclusion criteria included studies with (I) Non-adult population (< 18 years), (II) Patients undergoing cardiac surgeries, or (III) Observational, retrospective, or unpublished studies.

### Search strategy and data extraction

We systematically searched PubMed, Embase, and Cochrane Library databases from inception to July 2024, with the following search terms: "Hypotension Prediction Index", "HPI", "intraoperative hypotension", "hemodynamic management", "goal-directed therapy", "vasopressors", "postoperative hypotension", "mortality", "fluid administration", "blood products". No language restrictions were used. References from all included studies, previous systematic reviews and meta-analyses were also manually searched to identify any additional studies. Two authors (V.F., I.Q.) independently extracted data from the selected studies. A template was developed for data extraction of relevant items, including study details (first author, publication year, study design, sample size, type of surgery), participants baseline characteristics (population characteristics, age, sex, ASA physical status), intervention (HPI protocol), control (type of monitorization), and outcome measures. Disagreements were resolved by consensus. Other databases such as Web of Science and Scopus were not included due to overlap in indexed studies and feasibility constraints.

### Handling of missing data

Missing data were managed through sensitivity analyses and, when possible, by contacting study authors. If data remained unavailable, an available-case analysis was conducted to minimize bias. Studies with a high proportion of missing data were flagged for quality and risk of bias assessment.

### Endpoints

The outcomes were Time-Weighted Average (TWA) of Mean Arterial Pressure (MAP) < 65 mmHg, duration of MAP < 65 mmHg, Area Under the Curve (AUC) for MAP < 65 mmHg, hypotension per patient, colloids use, crystalloids use, noradrenaline use, phenylephrine use, and ephedrine use, as well as the incidence of Acute Kidney Injury (AKI), hospital length of stay, blood loss, and the number of hypotensive events.

### Risk of bias assessment

Two authors (A.T., L.B.) independently assessed the risk of bias. Disagreements were resolved with a third author (V.F.). The Cochrane Collaboration’s Risk of Bias-2 (RoB-2) tool was used to evaluate the risk of bias in randomized trials. RoB-2 has 5 domains, specifically selection, performance, detection, attrition, and reporting.^16^

Publication bias was assessed by funnel-plot analysis to evaluate the symmetric distribution of trials with similar weights. No quantitative assessment of small studies or publication bias was performed due to the small number of studies included in each individual outcome.^17^

### Sensitivity analyses

We performed leave-one-out sensitivity analyses for the primary outcomes to assess the impact of individual studies on the pooled estimates. Studies were sequentially excluded, and the meta-analyses recalculated to ensure the robustness of the findings. Although univariable meta-regression analyses were conducted, multivariable meta-regression was not performed due to the limited number of studies per covariate (κ < 10), which would increase the risk of overfitting.^15^

### Statistical analysis

We pooled Risk Ratios (RR) and Mean Differences (MD) with 95 % Confidence Intervals (95 % CI) for categorical and continuous outcomes, respectively. DerSimonian and Laird random-effects models were employed for all endpoints due to the heterogeneity in methodology and demographics across the individual studies.^18^^,^^19^ We assessed heterogeneity with I² statistics and Cochran *Q* test; p-values < 0.10 and I² > 25 % were considered significant for heterogeneity.^18^ All statistical analyses were performed using *R* software version 4.3.2 (R foundation, Vienna, Austria). Statistical analyses were performed using R Software, version 4.3.2 (R Foundation for Statistical Computing, Vienna, Austria).

### Trial sequential analysis

To evaluate whether the cumulative evidence had adequate statistical power, we performed a Trial Sequential Analysis (TSA) for the primary outcome. Our methodology included two-sided hypothesis testing, with a type I error set at 5 % and a type II error at 20 %. We established both conventional and Trial Sequential Monitoring Boundaries (TSMB) for the HPI and standard groups. The sequential analysis accounted for heterogeneity using a variance-based correction, and a random effects model was applied. A z-score curve was generated to assess the strength and reliability of the evidence. Additionally, we estimated the number of patients required in a meta-analysis to determine whether the intervention should be accepted or rejected. TSA enhances the robustness of findings by ensuring that conclusions are supported either when the sample size surpasses the required threshold or when the z-curves cross the TSMBs before reaching the necessary patient count.^20^

## Results

### Study selection and characteristics

**Figure 1:**
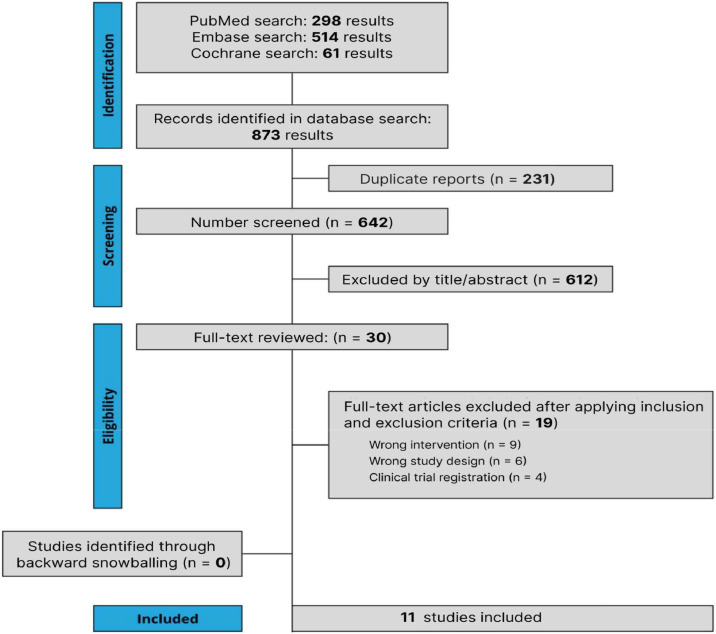
PRISMA flow diagram illustrating the selection process for studies included in the systematic review and meta-analysis***.***

In July 2024, the initial search identified 873 studies. After eliminating duplicates and applying the eligibility criteria, 31 studies were selected for full-text review, as illustrated in Figure 1.^10^, ^11^, ^12^, ^13^^,^^21^, ^22^, ^23^, ^24^, ^25^, ^26^, ^27^ A total of 11 studies met the inclusion criteria for the meta-analysis. The mean age of participants varied between 55 and 70.9 years. Overall, the baseline characteristics of the included studies were largely comparable, as presented in Table 1.

**Table 1:** Baseline characteristics of the included studies.

Study	N° of patients, HPI/ Stand	Male, HPI/ Stand ( %)	Age, years, HPI/ Stand	ASAI and II, HPI/ Stand ( %)	ASA III and IV, HPI/ Stand ( %)	BMI kg.m^-2^, HPI/ Stand	Surgery time HPI/ Stand (min)	Anesthesia time HPI / Stand (min)	Surgery Type	
									LPT HPI / Stand ( %)	LPS HPI / Stand ( %)
Wijnberge et al. 2020	31/29	68/45	68/62	80.65/93.10	19.35/6.90	24.2/24.7	256/259	302/300	61/45	7/17
Frassanito et al. 2023	30/30	0/0	55/59	80/ 83.33	20/16.67	23/22	N/A	298/305	50/80	50/20
Koo et al. 2022	35/33	31.4/48.5	64/63	100/100	0/0	N/A	207.9/208	N/A	100/100	0/0
Lai et al. 2024	30/30	76.7/76.7	60.17/23.3	36.7/59.7	36.7/50	22.2/21.7	517.5/491.4	N/A	N/A	N/A
Maheshwari et al. 2020	105/108	55.2/60.2	67/66	4.8/1.9	95.2/98.2	29/29	342/372	N/A	N/A	N/A
Murabito et al. 2022	20/20	50/60	69/ 70.5	45/50	55/50	25.3/25.6	207/237	N/A	N/A	N/A
Schneck et al. 2020	25/24	48/54	66/60	72/92	28/8	28.5/27.9	144/148	190/195	N/A	N/A
Sribar et al. 2023	40/40	65/60	60/59	72/92	28/8	26/28.4	81/82	169/185	N/A	N/A
Tsompa et al. 2021	49/50	53/58	66/70	86/90	14/10	27.7/27.4	207/207	240/240	N/A	N/A
Yoshikawa et al. 2024	30/30	40/47	68/67	93/93	7/7	22/21	272/316	380/316	43/43	57/57

ASA, American Society of Anesthesiologist Physical Status Classification System; BMI, Body Mass Index; LPT, Laparotomy: LPS, Laparoscopy.

### Hypotensive outcomes

**Figure 2:**
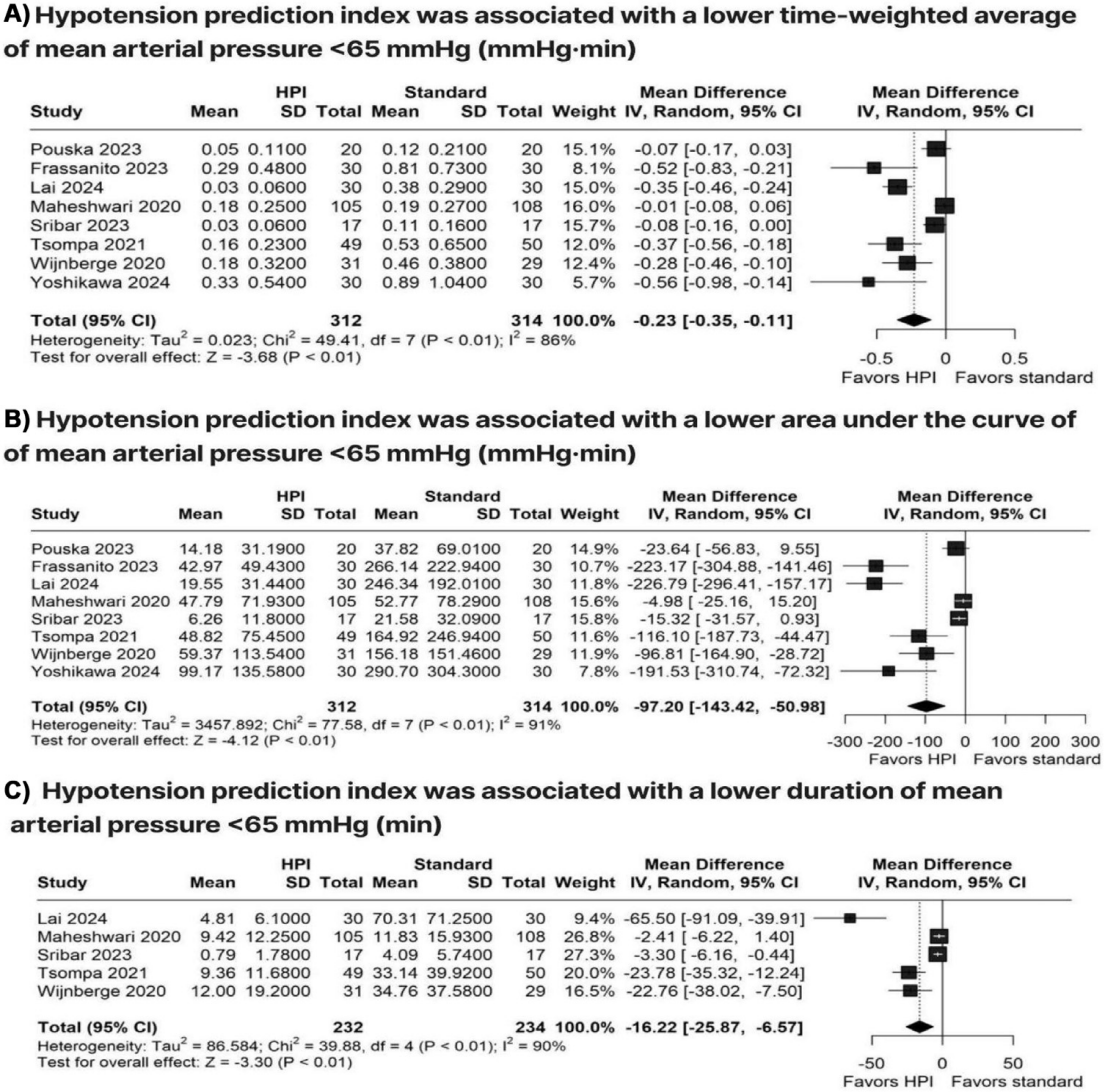
Forest plots comparing HPI-guided versus standard monitoring for (A) Time-Weighted Average (TWA) of MAP < 65 mmHg, (B) Area Under the Curve (AUC) for MAP < 65 mmHg, and (C) duration of MAP < 65 mmHg. MAP, Mean Arterial Pressure; TWA, Time-Weighted Average; AUC, Area Under the Curve.

**Figure 3:**
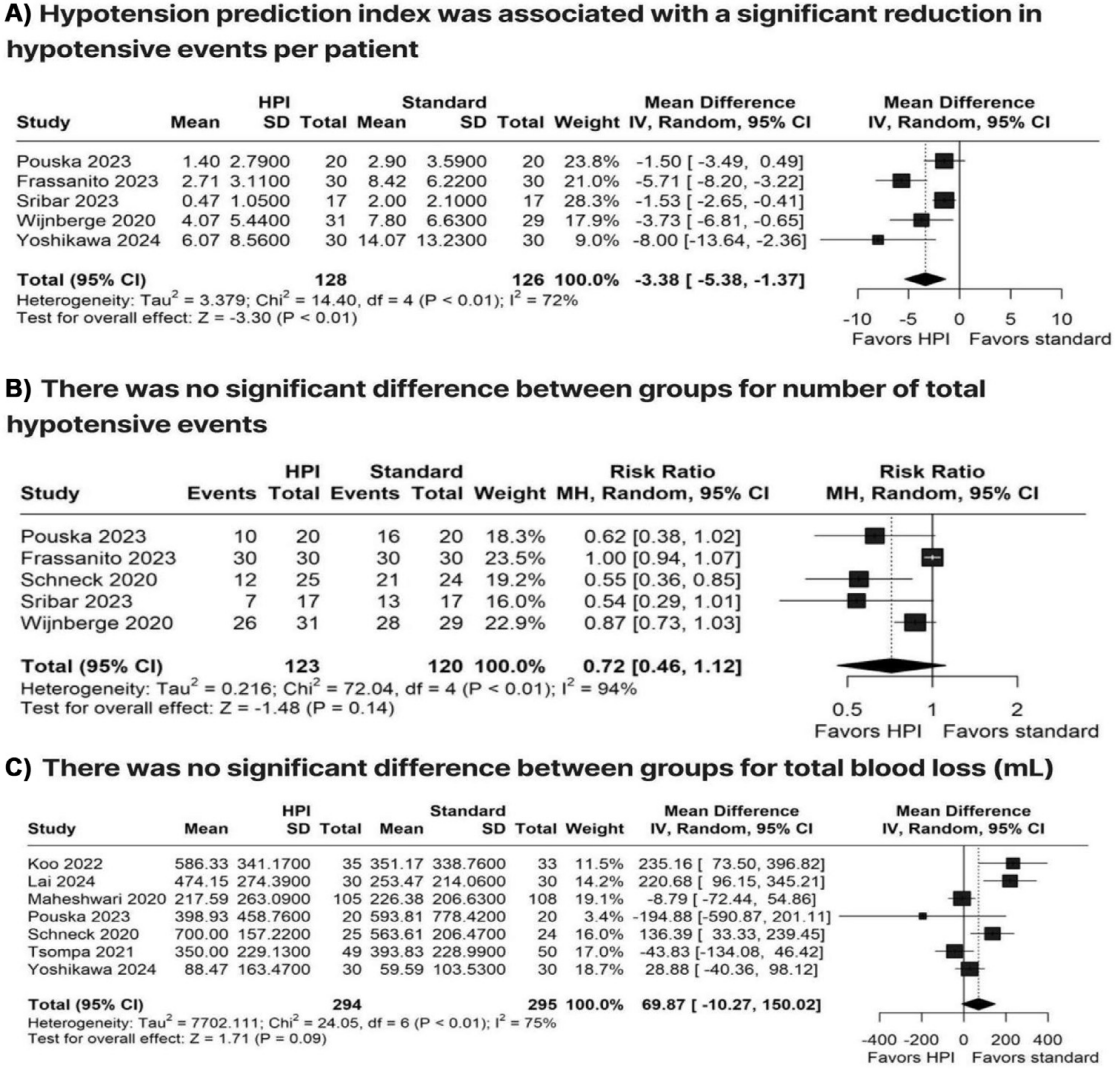
Forest plots showing (A) number of hypotensive episodes per patient, (B) number of hypotensive events, and (C) intraoperative blood loss.

The use of HPI was associated with significantly lower TWA < 65 mmHg (MD = −0.23 mmHg; 95 % CI −0.35 to −0.1; *p* < 0.01; I² = 86 %; Figure 2A) and lower AUC < 65 mmHg (MD = −97.20 mmHg.min^-1^; 95 % CI −143.42 to −50.98; *p* < 0.01; I² = 91 %; Figure 2B) compared with the standard group. Additionally, HPI resulted in a reduced duration of MAP < 65 mmHg (MD = −16.22 min; 95 % CI −25.87 to −6.57; *p* < 0.01; I² = 90 %; Figure 2C) and a decrease in hypotension per patient (MD −3.38; 95 % CI −5.38 to −1.37; *p* < 0.01; I² = 72 %; Figure 3A). However, no significant differences were observed between the groups regarding the number of hypotensive events (RR=0.72; 95 % CI 0.46 to 1.12; *p* = 0.14; I² = 94 %; Figure 3B) or blood loss (MD = 69.87 mL; 95 % CI −10.27 to 150.02; *p* = 0.09; I² = 75 %; Figure 3C).

### Drugs

**Figure 4:**
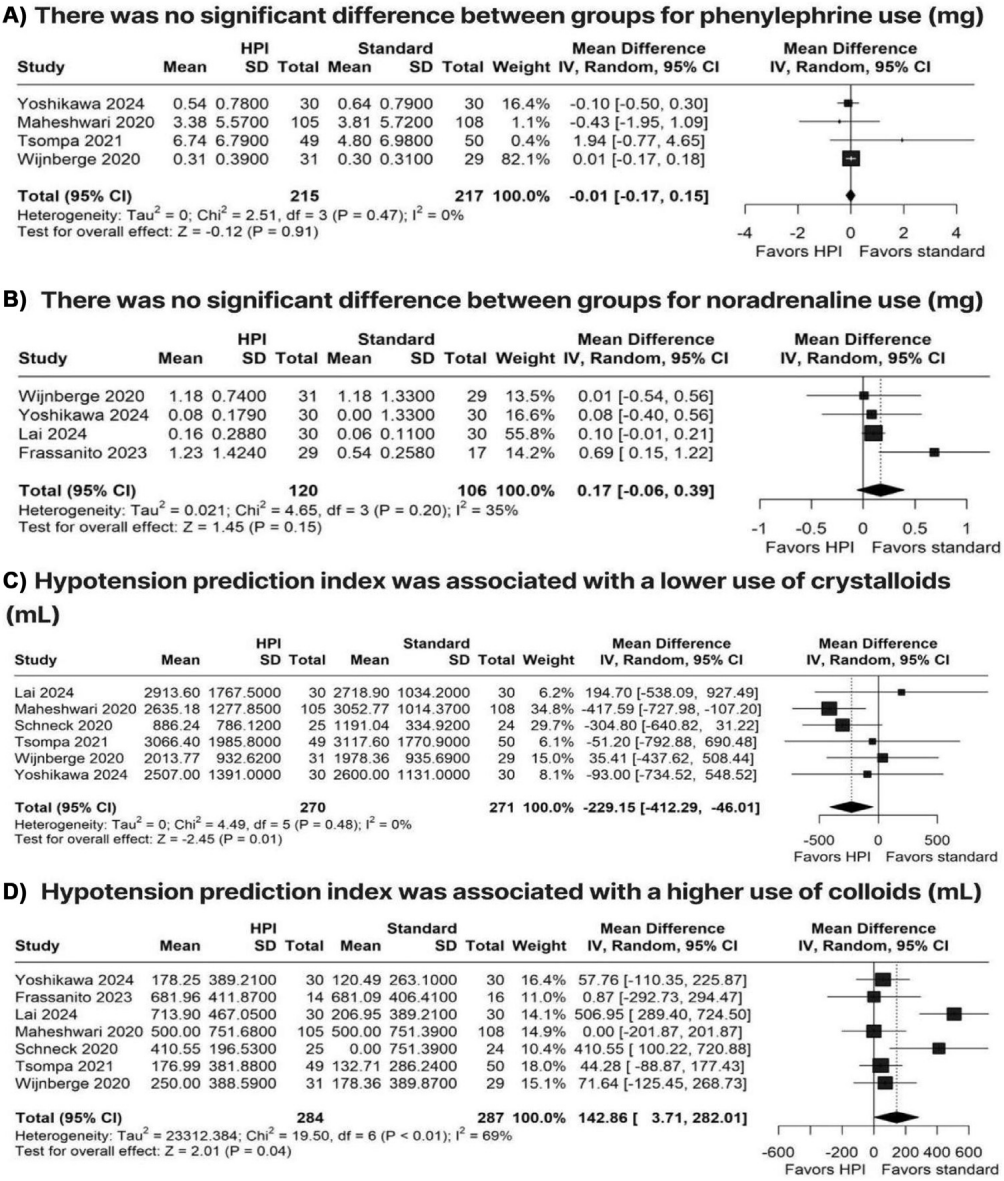
Forest plots comparing (A) phenylephrine use, (B) noradrenaline use, (C) crystalloid volume administered, and (D) colloid volume administered in HPI-guided versus standard care groups. HPI, Hypotension Prediction Index.

There was no significant difference between HPI and standard care in the use of phenylephrine (MD = −0.01 mg; 95 % CI −0.17 to 0.15; *p* = 0.91; I² = 0 %; Figure 4A) or noradrenaline (MD = 0.17 mg; 95 % CI −0.06 to 0.39; *p* = 0.15; I² = 35 %; Figure 4B) intraoperatively. HPI was associated with a lower use of crystalloids (MD = −229.15 mL; 95 % CI −412.29 to −46.01; *p* = 0.01; I² = 0 %; Figure 4C) and an increased use of colloids (MD = 142.86 mL; 95 % CI 3.71 to 282.01; *p* = 0.04; I² = 69 %; Figure 4D).

### Acute kidney failure

Incidence of AKI (RR = 0.81; 95 % CI 0.48 to 1.36; *p* = 0.42; I² = 0 %; Supplementary Fig. S1) was similar between patients who underwent surgery with HPI and patients who underwent surgery with the standard monitorization.

### Hospital length of stay

There were no significant differences between groups for hospital length of stay (MD = 0.12 days; 95 % CI −0.49 to 0.74; *p* = 0.69; I² = 0 %; Supplementary Fig. S2).

### Sensitivity analyses

Leave-one-out sensitivity analysis for the outcome of TWA < 65 mmHg revealed consistent results after omitting each individual study. The results for the sensitivity analysis are presented in Supplementary Figure S3.

### Trial sequential analysis

TSA showed that there is sufficient evidence for the reduction in TWA < 65 mmHg with HPI when compared to standard monitoring, as the cumulative z-curve crosses both the TSMB and the required information size (Supplementary Fig. S4).

### Risk of bias assessment

Among the 11 included RCTs, 10 were classified as having an overall low risk of bias.^10^, ^11^, ^12^, ^13^^,^^21^, ^22^, ^23^, ^24^, ^25^, ^26^, ^27^ However, one study was identified as having some concerns regarding the randomization process and was rated as presenting an overall moderate risk of bias (Supplementary Fig. S5).^13^

The funnel plot for TWA < 65 mmHg (Supplementary Fig. S6) showed no apparent asymmetry, suggesting no strong evidence of publication bias. This finding was further supported by Egger’s test, which indicated no significant small-study effects.

## Discussion

In this systematic review and meta-analysis of 11 Randomized Controlled Trials (RCTs), we evaluated the effectiveness of the Hypotension Prediction Index (HPI) compared to standard monitoring in patients undergoing non-cardiac surgeries. Our findings demonstrated that HPI significantly reduced both the incidence and duration of Intraoperative Hypotension (IOH) across diverse surgical contexts. Specifically, HPI was associated with reductions in the Time-Weighted Average (TWA) of Mean Arterial Pressure (MAP) below 65 mmHg, the Area Under the Curve (AUC) for MAP below 65 mmHg, the number of hypotensive events per patient, and crystalloid administration (approximately 230 mL less compared to standard care). Conversely, no significant differences were identified regarding adverse events such as hypertension or Acute Kidney Injury (AKI) between HPI-guided and standard care groups.^10^, ^11^, ^12^, ^13^^,^^21^, ^22^, ^23^, ^24^, ^25^, ^26^, ^27^

Technological advancements in Artificial Intelligence (AI) increasingly transform clinical practice by enabling real-time analysis of patient data to anticipate adverse outcomes. HPI leverages AI to analyze arterial waveforms, predicting potential hemodynamic instability up to 15 min in advance, thereby shifting intraoperative management from reactive to proactive.^28^, ^29^, ^30^ Maheshwari et al. evaluated the algorithm in adult patients over 45 years of age undergoing moderate- to high-risk non-cardiac surgery, initially finding no significant difference in hypotension duration unless clinical interventions were actively executed following HPI alerts, highlighting the importance of prompt responses to predictive warnings.^25^ Similarly, Wijnberge and colleagues, in the HYPE trial involving non-cardiac surgical patients, confirmed significant reductions in hypotensive episodes associated with HPI use.^22^ These findings collectively underscore the necessity of timely interventions following AI-based predictions, reinforcing the clinical value of integrating HPI technology into routine practice.

Our analysis consistently demonstrated that HPI-guided management significantly reduced hypotensive episodes during surgery, corroborating prior studies that also reported significantly lower TWA of MAP below 65 mmHg compared to standard care.^22^^,^^25^ Clinically, even brief episodes of hypotension are linked with increased risks of acute kidney injury, myocardial ischemia, and neurological complications.^3^^,^^6^^,^^31^ Gregory et al. previously showed that incremental decreases in MAP correlate with significantly increased risks for postoperative adverse events.^32^ Thus, the observed reduction of approximately 16.22 min in hypotension duration with HPI use is clinically relevant, potentially decreasing cumulative organ hypoperfusion and minimizing the risks associated with IOH, although these specific outcomes were not statistically significant in our meta-analysis.

Despite these promising findings, our meta-analysis showed substantial heterogeneity (I² frequently above 70 %), potentially due to differences in protocols, anesthetic techniques, surgical populations, and varying operational definitions of IOH across studies. Future subgroup analyses or meta-regression could clarify sources of heterogeneity, helping to identify specific patient populations or surgical contexts that benefit most from HPI-guided management.

Additionally, although our analysis revealed significant reductions in crystalloid administration, there was no observed significant difference in vasopressor use, and clinical outcomes such as AKI and hospital Length of Stay (LOS) remained unaffected. This absence of significant differences in relevant clinical outcomes could be attributed to the high heterogeneity and variability in patient populations and surgical scenarios included in our analysis. Moreover, in the context of Enhanced Recovery Protocols (ERAS), expecting significant improvements in outcomes from a single intervention, such as HPI-guided hypotension management, may be overly simplistic, given the multifactorial nature of postoperative complications.

Rather than functioning as a standalone solution, HPI can be effectively integrated into existing goal-directed therapy protocols, complementing other hemodynamic monitoring tools to enhance intraoperative management.^33^ This proactive approach, when combined with fluid and vasopressor management strategies, can optimize tissue perfusion, reduce the risk of organ dysfunction, and ultimately improve patient outcomes.^33^ Additionally, HPI can provide a probability score ranging from 0 to 100, indicating the likelihood of hypotension occurring within the next 5, 10, 15 min.^34^ This enables timely interventions before significant drops in MAP occur. In our meta-analysis, the HPI was associated with a reduction of 16.2 min in time spent with a MAP < 65 mmHg. This finding suggests that HPI not only reduces the occurrence of hypotension but also shortens its duration when it does occur, potentially reducing the cumulative harm from extended periods of low blood pressure.^33^

Gregory et al. showed that for every absolute maximum decrease in MAP, the odds of a major adverse event within 30 days post-surgery increased by 12 % for MAP ≤ 75 mmHg, 17 % for MAP ≤ 65 mmHg, and 26 % for MAP ≤ 55 mmHg.^32^ Despite these well-established associations between IOH and adverse events, our analysis did not show significant differences for AKI between the HPI and standard care groups.^32^ However, it is essential to consider the different patient profiles and surgical contexts across the included trials. Future research should focus on stratifying patient populations to determine whether HPI is more beneficial in specific subgroups, particularly those at higher risk for hemodynamic instability.

A key challenge in utilizing HPI lies in striking the right balance between preventing hypotension and avoiding overtreatment, which can result in hypertension or unnecessary fluid administration. An observational study found that patients who underwent surgery with HPI monitors had a significantly higher number of hypertensive episodes.^23^ Although our meta-analysis demonstrated that HPI-guided therapy significantly reduced crystalloid use, we did not find a significant difference in vasopressor administration. These findings underscore the importance of careful calibration of interventions based on HPI predictions to avoid unnecessary fluctuations in blood pressure and excessive therapeutic measures. Overcorrecting hypotension can lead to other hemodynamic disturbances, such as hypertension, which carries its own set of risks, including postoperative bleeding and cardiovascular stress.^35^

HPI represents a significant advancement in hemodynamic management, and its evolution is paving the way for non-invasive applications.^36^ Traditionally, HPI has relied on invasive arterial catheterization to obtain accurate arterial waveform data, which is essential for its predictive algorithm.^9^ Recent innovations in non-invasive arterial pressure monitoring systems, such as finger-cuff technologies, are broadening HPI’s clinical applicability. These non-invasive approaches demonstrate promising predictive accuracy (sensitivity and specificity of approximately 0.86 at 5 min prior to hypotension), overcoming previous limitations related to invasiveness and limited applicability highlighted by Hatib et al.^37^ By integrating these non-invasive monitoring techniques with predictive HPI algorithms, clinicians may achieve proactive and precise hemodynamic management across broader clinical scenarios, enhancing patient safety and outcomes without reliance on invasive procedures.^30^^,^^38^^,^^39^

### Strengths and limitations

A key strength of this meta-analysis is the comprehensive integration of recent literature evaluating HPI-guided management across varied clinical contexts, thus providing a robust synthesis of current evidence. Our analysis highlights the practical benefits of HPI implementation in reducing hypotension duration and crystalloid administration, outcomes directly linked to enhanced intraoperative management and potential clinical improvements. However, the study's hypothesis was founded on a relatively superficial exploration of existing literature concerning specific mechanisms by which HPI may influence clinical outcomes. Future studies would benefit from a deeper mechanistic understanding, clearly articulating the pathways through which HPI-guided intervention could reduce postoperative complications.

Additionally, significant heterogeneity among included trials presents limitations to the generalizability of our findings. Variability in patient populations, anesthetic practices, and definitions of hypotension contributed to the high heterogeneity observed. The limited feasibility of multivariable meta-regression further constrained our ability to explore effect modifiers. Future research using more granular subgroup analyses and robust multivariable models may help identify patients and clinical contexts that benefit most from HPI-guided management.

## Conclusion

In this systematic review and meta-analysis of adult patients undergoing non-cardiac surgeries, we found that the HPI significantly reduced the incidence and duration of IOH compared to standard monitoring. HPI was also associated with lower TWA of MAP < 65 mmHg and reduced use of crystalloids, without increasing vasopressor usage or causing adverse events.

## Declaration of competing interest

The authors declare that the research was conducted in the absence of any commercial or financial relationships that could be construed as a potential conflict of interest.

## Appendix Supplementary materials

Image, application 1

## Footnotes

Supplementary material associated with this article can be found in the online version at doi:10.1016/j.bjane.2025.844649.
