# Supplementary material for: Effectiveness of the hypotension prediction index in non-cardiac surgeries: a systematic review, meta-analysis and trial sequential analysis
Source: Braz J Anesthesiol. 2025 Jun 10;75(5):844649. doi: 10.1016/j.bjane.2025.844649 (PMC12271063; doi:10.1016/j.bjane.2025.844649)

**BJAN-D-25-00106**

**Supplemental Material:

Table of Contents**

**Supplementary Methods 1**: Search strategy

**Supplementary Figure S1** - Forest plot: acute kidney injury.

**Supplementary Figure S2** – Forest plot: hospital length of stay.

**Supplementary Figure S3** - Leave-one-out sensitivity analysis: time-weighted average of MAP < 65 mmHg

**Supplementary Figure S4**– Trial sequential analysis: time-weighted average (TWA) of MAP < 65 mmHg

**Supplementary Figure S5** - Risk of bias assessment (RoB2).

**Supplementary Figure S6** – Funnel plot: Time-weighted average of MAP < 65 mmHg.

***Supplementary Methods 1 Search strategy.***

| ("Hypotension Prediction Index" OR "HPI") AND ("intraoperative hypotension" OR "hemodynamic management" OR "goal directed therapy" OR "vasopressors" OR "postoperative hypotension" OR "mortality" OR "fluid administration" OR "blood products" OR "postoperative complications") |
| --- |

***Supplementary Figure S1*** ***Incidence of AKI****.*

There was no significant difference in AKI incidence between the HPI and standard groups (RR=0.81; 95% CI 0.48 to 1.36), with low heterogeneity (I²=0%).

*
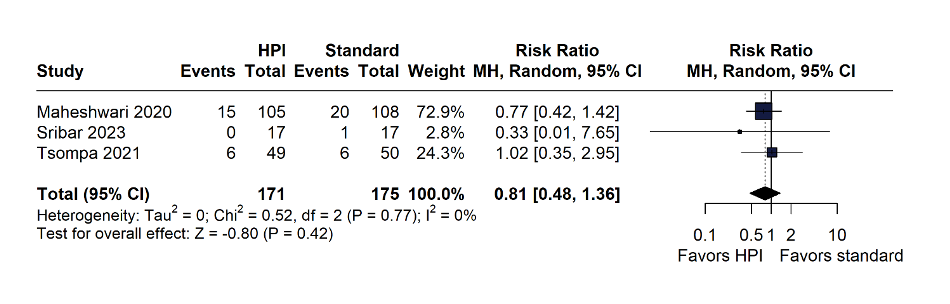
*

***Supplementary Figure S2 Hospital Length of Stay.***

The forest plot shows the mean difference in hospital length of stay between the HPI and standard groups. There was no significant difference between the groups (MD = 0.12 days; 95% CI -0.49 to 0.74), and the heterogeneity was low (I² = 0%).


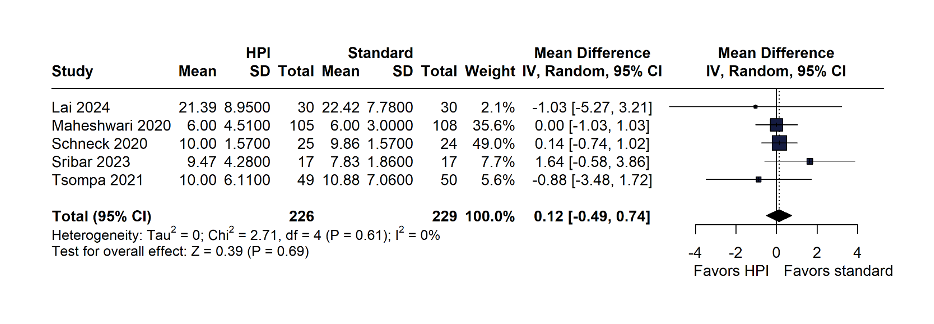


***Supplementary Figure S3 Sensitivity Analysis for Primary Outcome.***

Sensitivity analysis, using the leave-one-out method, showed consistent results for TWA < 65 mmHg (MD = -0.23; 95% CI -0.35 to -0.11; I²=86%) after omitting each individual study, confirming the robustness of the findings.

*
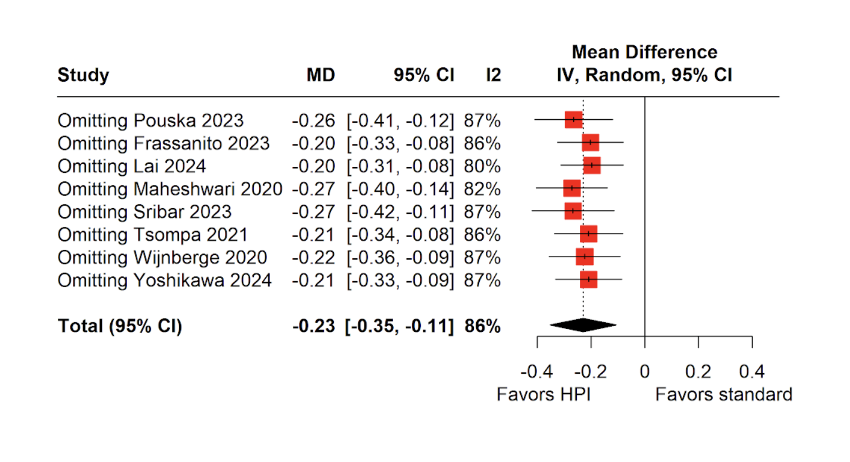
*

***Supplementary Figure S4 Trial Sequential Analysis (TSA).***

The TSA graph for TWA < 65 mmHg indicates that the cumulative evidence is sufficient to support the effectiveness of HPI in reducing hypotensive events. The cumulative z-curve crosses the conventional boundary as well as the required information size, confirming that the findings are robust and conclusive.


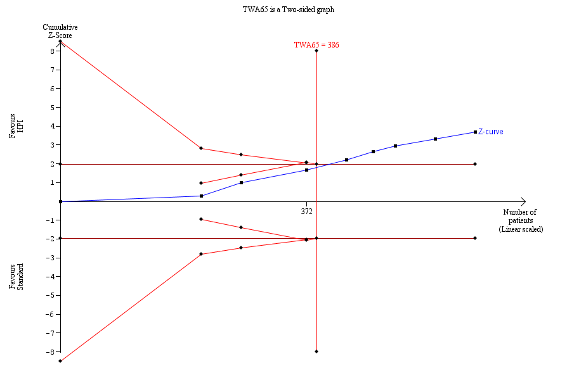


***Supplementary Figure S5 Risk of Bias Analysis.***

The risk of bias assessment using the RoB-2 tool showed that ten out of eleven studies had an overall low risk of bias, while one study (Frassanito 2023) presented some concerns due to the randomization process.

*
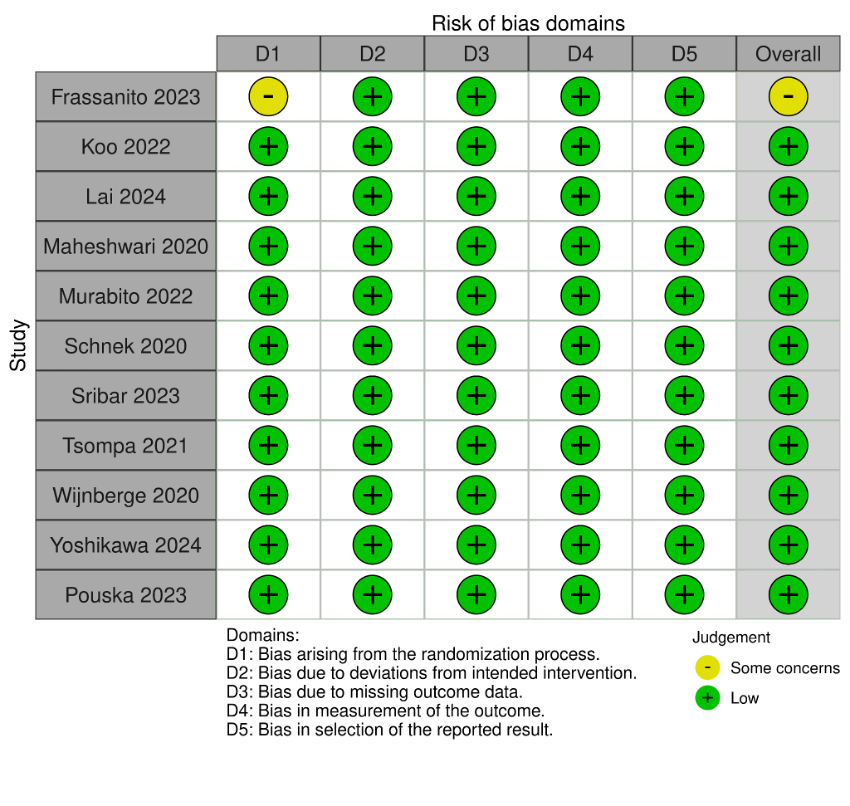
*

***Supplementary Figure S6 Funnel Plot for Publication Bias (TWA < 65)*.**

The funnel plot shows the distribution of individual studies based on the standard error and mean difference. Most studies are symmetrically distributed, suggesting no significant publication bias. However, a few outliers, like Yoshikawa 2024 and Frassanito 2023, are positioned asymmetrically, indicating potential bias.


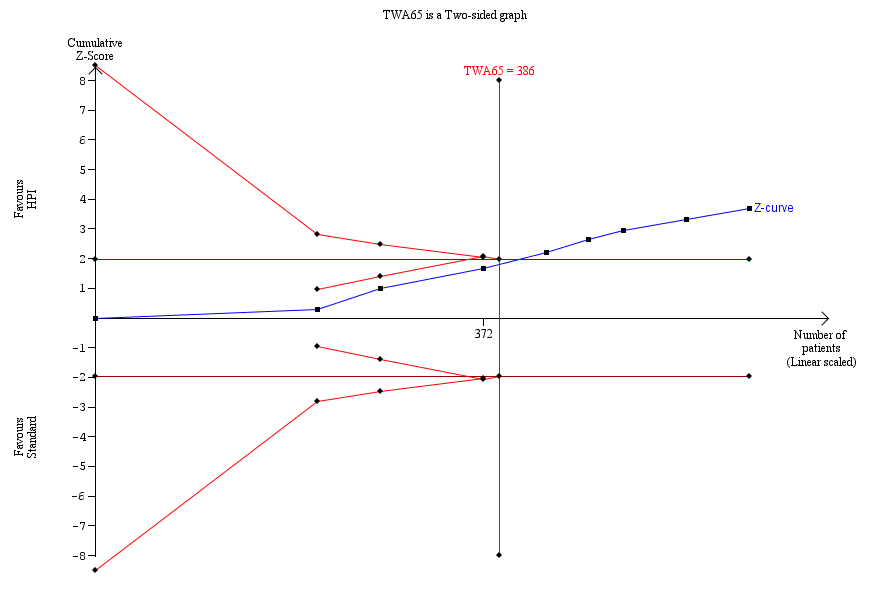

Supplement: Supplementary file 1 [file mmc1.docx]
